# Six-Minute Walk Test in Renal Failure Patients: Representative Results, Performance Analysis and Perceived Dyspnea Predictors

**DOI:** 10.1371/journal.pone.0150414

**Published:** 2016-03-16

**Authors:** Maja Bučar Pajek, Ivan Čuk, Bojan Leskošek, Gregor Mlinšek, Jadranka Buturović Ponikvar, Jernej Pajek

**Affiliations:** 1 Faculty of Sport, University of Ljubljana, Ljubljana, Slovenia; 2 Department of Nephrology, University Medical Centre Ljubljana, Ljubljana, Slovenia; University of Leicester, UNITED KINGDOM

## Abstract

**Objectives:**

Six-minute walk test in dialysis population hasn’t been consistently evaluated for the isolated impact of renal failure and other predictive factors. We measured six-minute walk distance in patients representative for low level of comorbidity and searched for potentially modifiable predictive factors of performance and dyspnea.

**Methods:**

This was a cross-sectional study with hemodialysis patients (N = 90) and control subjects (N = 140). Main outcome measures: six-minute walk test distance and dyspnea severity using the 10-item Borg scale.

**Results:**

Median distance decreased from 600m below the 6^th^ decade to 420m in the 8^th^ decade of age. Dialysis dependence predicted 101.5m shorter distance in the adjusted model that explained 70% of variability in results. Adjusted for significant covariates of age, height and spontaneous gait speed, fat mass (but not lean body mass) and serum total iron binding capacity were significantly associated with distance (95% CI for B coefficients -4.6 to –1.4 m/kg and 0.1 to 5 m/μmol/l, respectively). Serum total iron binding capacity as an explanatory variable was superior to C-reactive protein and albumin. Dialysis dependence, odds ratio (OR) 2.97 (1.11–7.94), spontaneous gait speed, OR 0.08 (0.02–0.41), rate-pressure product, OR 1.15 (1.08–1.23) and hemoglobin, OR 0.95 (0.92–0.98) predicted dyspnea in the adjusted model.

**Conclusions:**

Renal failure without the confounding effect of comorbidity is a significant negative predictor of performance at six-minute walk test and perceived level of dyspnea. Body fat mass and serum total iron binding capacity are the main potentially modifiable predictors of performance, total iron binding capacity being superior to C-reactive protein and albumin. Although hemoglobin is not associated with test performance, it negatively predicts perceived shortness of breath.

## Introduction

Investigation of physical function in dialysis patients is gaining a widespread interest and recognition among dialysis clinicians and physical therapists. Research findings show that physical performance is one of the strongest predictors of survival in dialysis patients[1–3]. 6-minute walk test (6MWT) is a major physical tests in dialysis population [4]. Its value lies in the fact that it is a self-paced test of walking capacity and reflects the functional ability at daily physical activities which are mostly performed at submaximal level of exertion [5].

Although the 6MWT result is predictive of survival in dialysis patients [6], there is insufficient data about dependence of six-minute walk test on modifiable dialysis therapy related factors. Comorbid conditions such as pulmonary disease [7,8], heart failure [9], peripheral arterial disease [10] or neurological disease [11] may significantly shorten the walked distance, but so far the isolated influence of renal failure on test performance in dialysis patients without or with low level of comorbidity has not been explored. Representative results of six-minute walk test distance in such a sample would be very much needed to compare and benchmark the results of dialysis patients measured in various clinical settings. Additionally, age and body height are the two non-modifiable factors with a known influence in healthy subjects [12], however their influence was not consistently adjusted for in analyses of modifiable dialysis-therapy related factors. The relative importance of factors such as anemia, sarcopenia, malnutrition and inflammation is not well clarified but it should only be explored in an analysis adjusting for proper non-modifiable factors, such as age, height and comorbidity.

Limitations of physical activity and conditioning caused by dyspnea in dialysis patients are understudied. Discomfort associated with breathing contributes to exercise limitation in normal subjects and patients with cardiorespiratory diseases [13]. In dialysis patients dyspnea has been confirmed as a self-reported barrier to physical activity and training [14,15]. It is also one of the commonest symptoms in daily clinical practice. Up to now, no analysis of dyspnea predictors at physical function tests can be found for dialysis patients.

Since dialysis population suffers from high level of comorbid diseases which have a profound confounding effect on their physical performance and mortality, we executed a research in patients with low level of comorbidity and excluding advanced levels of comorbid conditions to address the so far unanswered questions—to what extent can the physical impediment of dialysis patients be attributed to the isolated impact of renal failure, how large is this influence and which routinely measured serum biomarkers of inflammation-malnutrition syndrome (C-reactive protein (CRP), albumin, serum total iron binding capacity (TIBC)) associate with this influence. We aimed to assess representative results of the 6MWT in dialysis patients with low level of comorbidity and to search for predictive factors of performance including key demographic, anthropometric and body composition covariates. Besides the 6MWT distance the predictors of perceived shortness of breath were also analyzed.

## Materials and Methods

### Study design and participant selection

We executed this work as a cross-sectional case-control study on a sample of hemodialysis patients and control subjects without renal disease to measure two main outcome measures: the 6MWT distance and perceived dyspnea severity. The measurements were done between July and December 2014 at the Faculty of Sports University of Ljubljana and included the sample of maintenance hemodialysis patients from 3 University Medical Centre Ljubljana’s outpatient dialysis units and seven other outpatient Slovenian dialysis units. Patients and control subjects were eligible for inclusion in the study if older than 18 years, able to walk with or without additional support and if they voluntarily granted the informed consent for inclusion in the study. The patients or control subjects were not included if any of following conditions was present: hospitalization or acute disease in the last 4 weeks before the study measurements, active malignant disease or chronic infection (i.e. tuberculosis, osteomyelitis), consequences of cerebrovascular accident (paresis or paralysis), heart failure of NYHA stage 3 or 4 or symptomatic angina pectoris Canadian Cardiovascular Society stage 2,3 or 4, chronic obstructive pulmonary disease stage 3 or 4, decompensated liver cirrhosis, symptomatic peripheral arterial obstructive disease, painful degenerative or inflammatory arthropathy with current use of anti-inflammatory or analgesic therapy or currently symptomatic psychiatric condition. Control subjects were recruited as a convenience sample of volunteers without known renal disease from a wide range of settings (work sites, schools, nursing homes, community centers for older adults). A demand for control subjects was the absence of history of renal disease or serum creatinine concentration below 133 μmol/l (1.5 mg/dl). Sample size was first calculated under the predictions of expected R^2^ value for multiple regression of the 6MWT distance of 0.4 [16], with desired statistical power level of 0.9, alpha level of 0.05 and with 7 predictors in the multivariate model. With these parameters, the calculated effect size f^2^ was 0.67 and the estimated required number of dialysis subjects was 36 [17]. However the target sample size for dialysis patients was increased to a minimum of 70 participants to allow at least 10 subjects in each decade from 20 to 80 years of age to give a better representation of results over the entire age span. We planned to recruit 140 control individuals to satisfy a 2:1 ratio with dialysis patients as the compensation for a convenience sampling of healthy controls.

### Study protocol and measurements

Dialysis patients were scheduled for study visit on non-dialysis days in the afternoon hours. A questionnaire to obtain demographic data, medical history, medications and socioeconomic data was applied, next anthropometric measurements and bioimpedance body composition analysis were done and finally spontaneous gait speed and the 6MWT were performed. Medical history, comorbid conditions and current dialysis related therapy were verified through inspection of health documentation and interview with attending dialysis physicians. Comorbid conditions were graded using the Davies comorbidity grade [18]. The concentrations of hemoglobin, albumin, CRP and TIBC were measured with routine laboratory methods at local laboratories.

Height was measured with a dedicated anthropometer (GPM, SiberHegner, Zurich, Switzerland) to the nearest 0.1 cm. Bioimpedance measurements were performed with a bioimpedance monitor (Fresenius Medical Care, Bad Homburg, Germany) as per manufacturer’s instructions. Body composition analysis provided measurements of total body water, extracellular water, over-hydration, lean and fat tissue mass. Spontaneous gait speed was measured by asking participants to walk at their usual pace over a marked 4-meter course, with the average time of two trials taken for gait speed calculation. The 6MWT was performed according to the American Thoracic Society’s guideline [5]. In short, the test was performed indoors on a 30m straight course. Participants graded their dyspnea and fatigue perception on the 10-scale Borg scale. At the end of the 6-minute period the distance was measured, immediate fatigue and dyspnea scores were reassessed and heart rate and blood pressure were measured.

### Statistical analyses

For statistical analyses baseline characteristics were summarized for control group and dialysis patients using means (SD) or medians (ranges) for continuous normally and non-normally distributed variables. Frequencies and percentages were used for categorical variables. General linear model analysis of variance (GLM ANOVA) was used for adjusted analyses of associations of independent explanatory predictor variables with the outcome variable of the 6MWT test distance. The effect size measure for predictor variables, the square of the partial correlation coefficient—partial Eta squared (which gives the proportion of variance in the dependent variable explained by the independent variable) was calculated and reported.

In the first stage of analyses we constructed an explanatory model of the 6MWT distance including all subjects with independent variables of age, sex, height, lean and fat tissue mass, spontaneous gait speed, comorbidity grade and dialysis dependence to test the isolated effect of renal failure and uremia. We did not rely on the univariate statistics to generate variables for adjusted analyses. Instead, we used published evidence (height, body composition) [16,19] and clinical experience (age, sex, spontaneous gait speed, comorbidity) to include the variables of interest. Here we additionally performed sensitivity analyses with same predictors (except dialysis dependence) in control subjects and dialysis patients separately, to test for robustness of findings in both parts of the sample. To analyze associations of malnutrition-inflammation biomarkers we constructed a separate GLM in the subsample of dialysis patients. We included all statistical significant factors from the general model and added pre-specified variables known to be associated with comorbidity (Davies comorbidity grade), anemia (hemoglobin concentration), inflammation (albumin, CRP, TIBC) and malnutrition (albumin, TIBC).

Dyspnea and fatigue predictors were analyzed by dividing the outcome variable in two groups: having a perceived dyspnea score (PDS) or fatigue score of none (0), very mild (1) or mild (2) defined the first outcome group and having a higher score (moderate—score 3 and higher scores, up to maximal score of 10) defined the second outcome group. This division criterion was chosen to divide the sample at the median value of PDS and this division corresponds well to the clinical relevance of symptom severity (up to mild or stronger). The probability to classify into one of both PDS groups was modelled using the binary logistic regression analysis. The independent variables were chosen to verify the impact of renal failure adjusted for factors associated with the 6MWT performance (age, height and fat tissue mass, spontaneous gait speed), motivation and exertion (pressure-rate product of post-test systolic blood pressure and heart rate) and clinical causes of dyspnea (comorbidity grade, anemia and over-hydration). Same independent variables were used for fatigue analysis. As a sensitivity analysis we repeated the model with exclusion of participants with hemoglobin level below 110 g/l (representing lowest quartile of dialysis patients most probable to develop the symptoms of dyspnea). Additional sensitivity analysis was done with use of the difference in PDS after and before the 6MWT as an outcome variable. Here we used binary logistic regression to model the probability of having a rise of PDS above or below the median difference.

Analyses were done using the IBM SPSS statistics application version 22 (IBM Corporation, USA). Adjusted analyses were performed by entering all independent variables of interest simultaneously, no stepwise methods were executed. The probability level of <0.05 was considered statistically significant. The study was approved by the Slovenian Medical Ethics Committee (document No. 125/05/14). All participants gave written informed consent to inclusion in the study.

## Results

**Fig 1:**
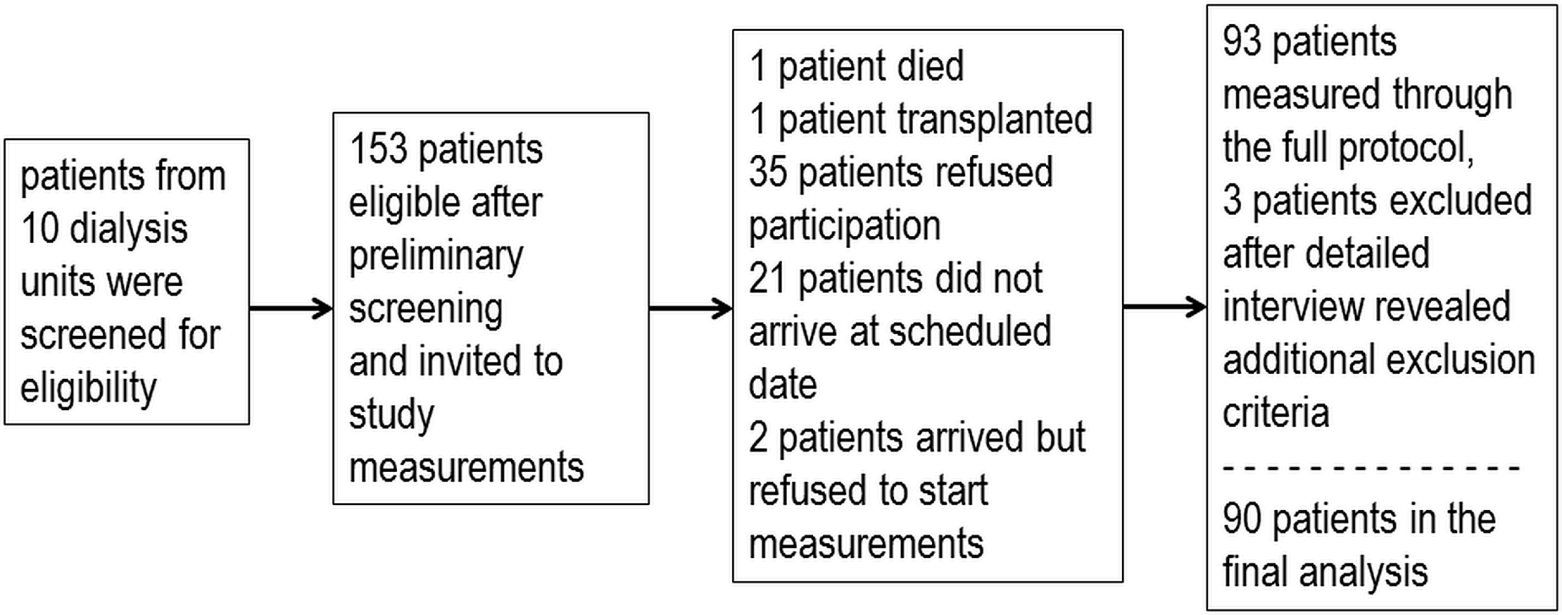
Flow of patients with end-stage renal disease through recruitment process. We also invited 180 community dwelling subjects to participation and 140 of them responded and performed the full study protocol. All participants were of European (white) descent.

In the final analysis we have included 90 hemodialysis patients and 140 control subjects. The flow of participants through recruitment process is shown in the Fig 1.

The demographic and clinical characteristics of controls and dialysis patients are shown in the Table 1.

**Table 1:** Demographic and clinical characteristics of subjects.

Parameter	Control group*^,^†	Patients on HD*^,^†	p
Sex (N male (%)/N female (%))	59 (42.1)/81 (57.9)	61 (67.8)/29 (32.2)	<0.001
Age (years)	51.7 (16.3)	55.2 (16)	0.12
Serum creatinine (μmol/l)	62 (12)	877 (232)	<0.001
Haemoglobin (g/l)	141 (12)	119 (12)	<0.001
Serum Albumin (g/l)	44.7 (2.5)	41.2 (3.6)	<0.001
BMI	26 (4.7)	26.1 (4.1)	0.51
Davies comorbidity grade 0/1/2 (N (%))	127 (90.7)/13 (9.3)/0	47 (52.2)/37 (41.1)/6 (6.7)	<0.001
Selected dialysis characteristics (median, range)			
Time interval from last HD session in hours	N/A	25 (9.5–42)	N/A
Years of dialysis treatment	N/A	4.6 (0–37.2)	N/A
Hours of dialysis per week	N/A	14 (8–23)	N/A
CRP (mg/l)	N/A	3 (3–30)	N/A
Serum phosphate (mmol/l)	N/A	1.5 (0.8–2.7)	N/A
Serum cholesterol (mmol/l)	N/A	4.1 (2.4–7)	N/A
TIBC (μmol/l)	N/A	42.7 (29.4–60.8)	N/A

BMI, body mass index; HD–hemodialysis; N/A–not applicable; TIBC, total iron binding capacity.

*Data are presented as mean (SD) or median (range) if not stated otherwise.

†Number of cases with missing values for patients on HD: 2 for CRP, 4 for cholesterol, 2 for TIBC, otherwise the dataset was complete. Number of cases with missing values for control subjects: 34 for albumin, 34 for creatinine, 31 for hemoglobin, otherwise the dataset was complete.

**Fig 2:**
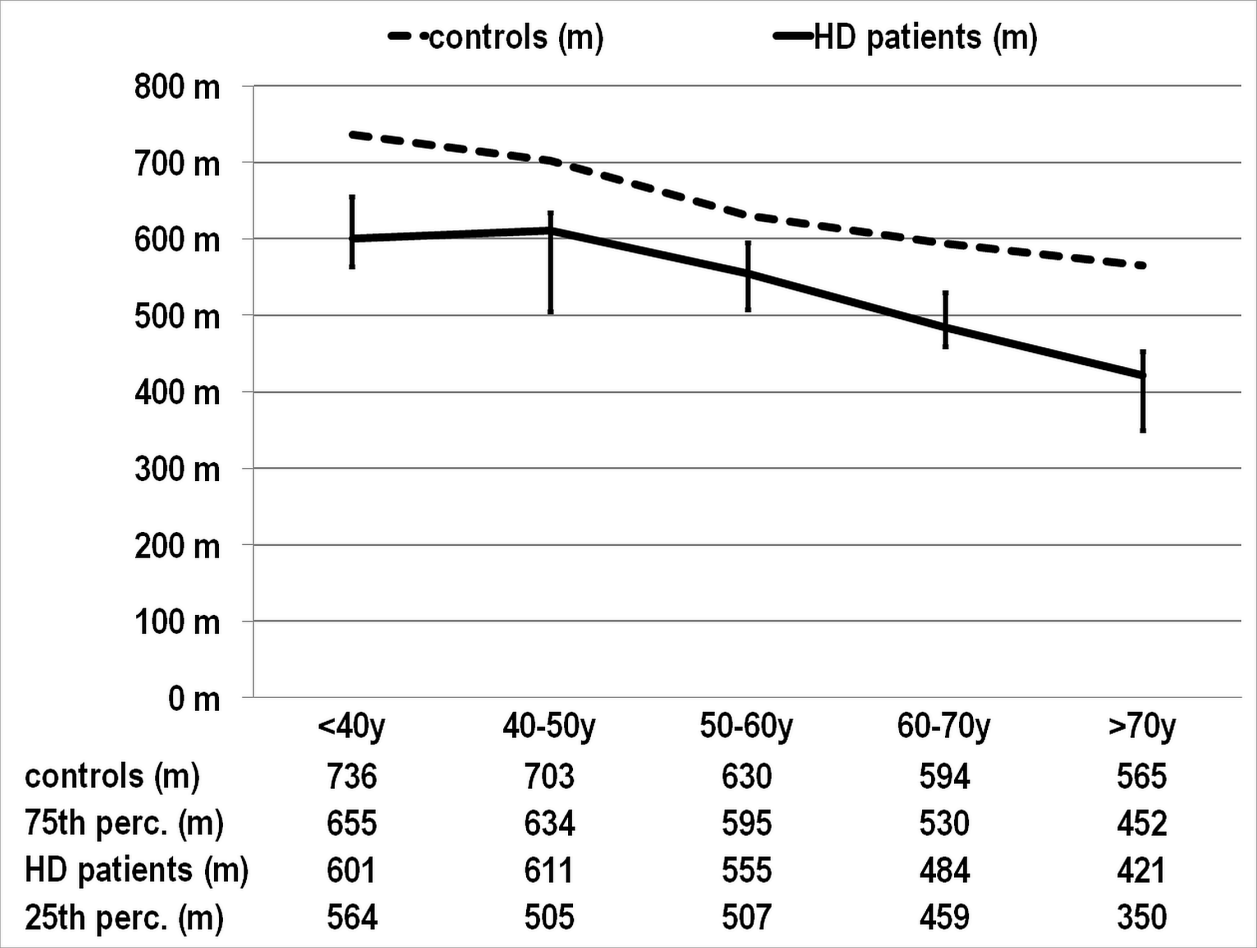
The results of 6-minute walk test by age group. Median values for dialysis patients–solid line and controls–dashed line. Interquartile range for dialysis patients is marked by error bars. 25^th^, 75^th^ percentile and median values for dialysis patients are presented below the chart together with median values for control subjects.

The results of the 6MWT divided by age groups are shown in the Fig 2. It can be seen that dialysis patients had lower performance across all age groups.

### Comparison of dialysis patients and control subjects to analyze the impact of renal failure

The analysis of predictive parameters of the 6MWT distance is shown in the Table 2. Tested variables were age, sex, height, spontaneous gait speed, lean body mass, fat body mass and end stage renal failure–dialysis dependence.

**Table 2:** Association of the 6MWT with renal failure adjusted for demographic and anthropometric variables (ANOVA, general linear model).

Parameter	Unadjusted analysis		Adjusted analysis*			
	B	p	B (SE)	95% C.I. for B	Partial Eta^2^	p
Age (years)	-4.2	<0.001	-2.1 (0.3)	-2.8 to -1.4	0.15	<0.001
Sex (female)	-14.9	0.33	14.2 (41)	-66.6 to 95	0.001	0.73
Body height (cm)	4.7	<0.001	3.3 (0.8)	1.8 to 4.9	0.08	<0.001
Spontaneous gait speed (m/s)	216.8	<0.001	92 (22.6)	47.7 to 136.5	0.07	<0.001
Lean tissue mass (kg)	5	<0.001	0.4 (0.9)	-1.3 to 2	0.001	0.68
Fat tissue mass (kg)	-4.3	<0.001	-3.2 (0.5)	-4.2 to -2.2	0.16	<0.001
Davies comorbidity grade 0 vs.2	169.3	<0.001	15.7 (39.8)	-62.8 to 94.1	0.001	0.69
Davies comorbidity grade 1 vs.2	69.9	0.13	11.6 (30.7)	-48.9 to 72.1	0.001	0.71
Renal failure	-117.3	<0.001	-101.5 (26)	-152.9 to -50.1	0.07	<0.001

N = 230 (140 control subjects and 90 dialysis patients)

* model R^2^ = 0.7, adjusted R^2^ = 0.68, intercept = 98.5 m. Significant negative coefficients B denote that physical performance worsens with increasing age, fat tissue mass and the presence of renal failure; significant positive coefficients B denote that physical performance improves with increasing height and spontaneous gait speed.

The adjusted analysis has shown that dialysis patients have 101.5 m shorter results compared to controls. The results of this model were robust and unchanged when tested separately for dialysis patients and controls: age, height, spontaneous gait speed and fat tissue mass remained significant independent predictors of the 6MWT. They explained 66% and 57% of variance in adjusted models for dialysis patients and control subjects, respectively (exact results are shown in S1 and S2 Tables).

### Serum biomarkers predictive of 6-minute walk test result in dialysis patients

In dialysis patients we tested parameters possibly related to the 6-minute walk test result due to their clinical significance as markers of comorbidity, anemia, malnutrition and inflammation: Davies comorbidity grade, hemoglobin, albumin, CRP and TIBC. The results are shown in the Table 3.

**Table 3:** Analysis of medical variables as predictors of 6-minute walk test result in dialysis patients.

Parameter	Unadjusted analysis		Adjusted analysis*			
	B	P	B (SE)	95% C.I.	Partial Eta^2^	p
Age	-4.4	<0.001	-3.1 (0.5)	-4.1 to -2	0.31	<0.001
Body height (cm)	4	0.001	3.9 (0.9)	2.1 to 5.6	0.21	<0.001
Fat tissue mass (kg)	-10.9	<0.001	-3 (0.8)	-4.6 to –1.4	0.16	<0.001
Spont. gait speed (m/s)	269.9	<0.001	111 (37.7)	36.4 to 186.5	0.11	0.004
Davies comorbidity grade 0 vs.2	105	0.02	37.2 (31.3)	-25.2 to 99.7	0.02	0.24
Davies comorbidity grade 1 vs.2	42	0.35	22.5 (31.1)	-39.4 to 84.4	0.01	0.47
TIBC (μmol/l)	0.5	0.78	2.5 (1.2)	0.1 to 5	0.05	0.04
Albumin (g/l)	-1.9	0.55	-4.8 (3.1)	-11 to 1.4	0.03	0.13
Haemoglobin (g/l)	1.3	0.15	0.6 (0.6)	-0.5 to 1.8	0.02	0.28
CRP (mg/l)	-54	0.04	-0.1 (1.6)	-3.2 to 3	0	0.93

General linear model ANOVA

*N = 86, adjusted analysis model R^2^ = 0.7, adjusted R^2^ = 0.66, intercept B = -39.9 m. Significant negative coefficients B denote that physical performance worsens with increasing age and fat tissue mass; significant positive coefficients B denote that physical performance improves with increasing height, spontaneous gait speed and TIBC.

In the adjusted analysis shown in the Table 3 we kept the previous independent significant predictor variables from the analysis shown in the Table 2: age, height, spontaneous gait speed and fat tissue mass. Of the theoretically plausible and potentially modifiable predictive clinical variables added, fat mass and serum TIBC remained significantly associated with physical performance.

### Predictors of shortness of breath

PDS above 2 (corresponding to Borg scale dyspnea levels above mild) was present in 48% and 44% of dialysis and control subjects immediately after the 6-minute walk, respectively. PDS of 2 was also the sample median. The probability of having a PDS above 2 (more than mild) was modelled using the logistic regression analysis. In the model we included theoretically plausible variables as explained in the methods section. The model is shown in the Table 4.

**Table 4:** Logistic regression analysis model of the probability of having a more than mild shortness of breath.

Parameter	95% C.I. for odds ratio			p
	Lower	Odds ratio	Upper	
Age (years)	0.99	1.01	1.04	0.44
Spontaneous gait speed (m/s)	0.02	0.08	0.41	0.002
Rate-pressure product/1000	1.08	1.15	1.23	<0.001
Hemoglobin (g/l)	0.92	0.95	0.98	0.001
Fat tissue mass (kg)	0.97	1	1.03	0.98
Body height (cm)	0.99	1.03	1.07	0.14
Over-hydration (l)	0.68	0.91	1.22	0.52
Davies comorbidity grade 2 vs.0	0.07	0.56	4.59	0.59
Davies comorbidity grade 1 vs.0	0.07	0.63	5.59	0.68
Dialysis dependence	1.11	2.97	7.94	0.03

N = 199, Model R^2^ = 0.24 (Nagelkerke), Model χ^2^ = 39.4, p<0.001. More than mild shortness of breath corresponds to dyspnea Borg scale grade 3 or more.

Adjusted results show that renal failure is a statistically significant independent positive predictor of dyspnea at the 6MWT together with rate-pressure product (also a positive predictor), spontaneous gait speed and hemoglobin concentration (both negative predictors). When the analysis was redone excluding the dialysis patients with hemoglobin level below 110 g/l (representing lowest quartile of patients) the results of the model were essentially unchanged, with hemoglobin remaining a significant independent negative predictor of dyspnea level (S3 Table). The model results were similar when the outcome was a change in PDS (a pre-specified sensitivity analysis—see S4 Table).

PDS and fatigue scores were significantly correlated (Spearman’s rho 0.64, p<0.001). Similar to dyspnea, fatigue scores at the end of the 6MWT had a median score of 2 on the Borg scale (range 0–10). The probability of having a fatigue score above the median (more than mild) was modelled with the same predictor variables as for PDS, see Table 4. Spontaneous gait speed and hemoglobin remained significant negative predictors of fatigue. Odds ratios (OR) with 95% CI were 0.07 (0.01–0.39) and 0.95 (0.92–0.98) for gait speed and hemoglobin concentration respectively, p < 0.01. As opposed to PDS, fatigue was positively predicted by age and body height (OR 1.04 (1.01–1.07) and 1.05 (1–1.09) respectively, p<0.05) but not dialysis dependence.

## Discussion

In the present work we examined performance of patients with end-stage renal disease at the 6MWT. We searched for associations with the potentially modifiable clinical variables and analyzed predictors of shortness of breath. We posted narrow inclusion criteria to select a sample of patients with low burden of comorbidities (and adjusted for residual comorbidity) to isolate specific effects of uremia. Our key results have shown that when fully adjusted for these variables, living with end-stage renal failure is an independent condition to lower performance and predicts shortness of breath at physical exertion; measured results given by decades of age can be used as representative values for Caucasian dialysis patients with low level of comorbidity and we showed that body fat mass and serum TIBC are the main potentially modifiable clinical predictors of the 6MWT results. Notably, serum hemoglobin was not an independent predictor of the 6MWT distance, however it proved to be an independent predictor of shortness of breath even at levels currently judged in the target range for dialysis patients [20]. TIBC was superior to albumin and CRP as a malnutrition-inflammation associated serum biomarker and predictor of physical function in this low-comorbidity patient sample.

It is known from the earlier work in this field [16,21] that height is a crucial anthropometric variable positively associated with the 6MWT distance, however the studies in dialysis patients so far did not consistently analyze this parameter and sometimes body mass index was used instead [19,22]. In the important work by Martinson et al [19] mid-thigh muscle area and intra-abdominal fat area were significantly associated with the 6MWT distance, a result confirmed by our findings for fat mass. Lean body mass, on the contrary, although a variable closely associated with muscle mass, was not associated with the 6MWT result in our analysis. We investigated the correlation of lean body mass as measured in our sample with height and found a high and significant correlation (Pearson r = 0.74, p<0.001, with partial correlation coefficient controlling for body weight of 0.65, p<0.001). So the exclusion of height from the adjusted model may confound the effect of muscle mass. Indeed, when height was omitted from our adjusted model, lean tissue mass became a statistical significant positive predictor of the 6MWT distance (p = 0.01). Therefore, when properly adjusted for height, fat tissue mass and not lean/muscle tissue mass is a determinant of the 6MWT performance.

Higher fat mass [23] and less specifically higher BMI [24] have both been associated with better survival in dialysis patients. Poorer physical performance is a strong predictor of mortality in dialysis patients [1–3] and it seems counter-intuitive that we found an association between a higher fat mass (presumably associated with survival benefit) and poorer 6MWT performance. There is some data showing that the protective effect of high BMI is limited to those patients with high muscle mass and that high BMI patients with high body fat have increased and not decreased mortality [25]. On the other hand it is also plausible that adiposity may be associated with survival advantage independently of physical functioning, perhaps through association with lower levels of inflammation [26]. Interventional studies with reduction of adiposity (but with preservation of muscle mass) are needed to clarify the impact of such intervention—clearly beneficial in general population—on physical functioning and survival of dialysis patients.

Serum TIBC is a direct reflection of serum transferrin values and its measurement with a low-cost colorimetric assay is used to substitute for higher cost transferrin immunologic assay [27]. TIBC level is known to be lowered in malnutrition states and chronic illnesses of dialysis patients [28]. TIBC has been included in the malnutrition-inflammation score due to its great sensitivity for the frequent subclinical syndrome of malnutrition and inflammation in this population [29,30]. This is also the most probable mechanism through which TIBC associates with inferior physical performance. Role of decreasing TIBC as a marker of protein-energy wasting and inflammation was corroborated by establishing its independent association with mortality (lower values indicate higher risk) [31]. In our study TIBC was superior to albumin and CRP as a significant and independent positive predictor of the 6MWT result. Kono et al recently reported a finding of a negative association of TIBC with the 6MWT result [22], which is hard to explain in the light of aforementioned associations of lower TIBC levels with malnutrition-inflammation syndrome. Their finding may have been influenced by a smaller sample and the absence of important demographic and anthropometric covariates in the multivariate analysis. To our knowledge, this is the first report to show a significant and independent positive association of TIBC with the 6MWT performance in patients with end-stage renal disease and adds importance to regular monitoring of TIBC in this population.

In the analysis of perceived dyspnea, an important limiting factor of physical function of dialysis patients [14,15], dialysis dependence confirmed to be a significant independent predictor of dyspnea at the 6MWT. This effect was present in the analysis adjusted for classical dyspnea associated factors such as anemia, (over)hydration level and age. Interestingly, hemoglobin level was negatively associated with dyspnea and fatigue even at the target range for dialysis population. Although increasing the hemoglobin concentration above 130 g/l is currently not recommended due to higher risks for stroke, hypertension and vascular access thrombosis [20], our results support anemia treatment to values above the generally accepted target range of 100–115 (and up to 130) g/l for selected patients with dyspnea limiting their physical performance and exercise rehabilitation. Some evidence that increasing the hemoglobin above the generally accepted target range of 100–120 g/l may help to relieve dyspnea symptoms, already exists [32].

The strength of our study is a well selected patient sample with low levels of comorbidity (93% without or with a single comorbid condition of limited severity). Using additional adjustment for remaining comorbidity the isolated effects of uremia on physical performance could be investigated. Our adjusted model explained 70% of variability in the 6MWT in dialysis patients, which is superior to previous studies reporting predictive equations in healthy individuals (with explained variability in the range from 40 to 62%) [12,16,21]. This statistical model may therefore be used for prediction of the 6MWT result in dialysis patients. The drawback of the study is the limited external validity of the findings for non-white populations. Also, the dialysis patients that eventually completed the measurements could be positively biased towards a better general health with lower number of comorbidities, lower levels of depression, post-dialysis fatigue and shortness of breath as these are all barriers to physical activity in this population [14,15]. Our results are therefore mainly applicable to a low comorbidity subgroup of dialysis patients, such as those waitlisted for renal transplantation. This may limit the external generalizability of results although it does contribute to higher internal validity in examination of the sole effects of renal failure without comorbid influence. This is needed to judge the possible improvements in the physical performance that may be gained by future improvements in dialysis therapy such as dosage, time of treatment, convective therapies, novel purification therapies aimed at middle, large and protein bound uremic toxins that are not cleared well with current dialysis therapy [33]. Finally, control subjects were recruited as a convenience sample and were not randomly chosen from the population. This may have influenced the nature of observed differences between control and dialysis subjects although we tried to increase the number of control subjects to at least 2:1 ratio with dialysis patients to diminish the impact of a possible selection bias in the control subjects.

## Conclusions

In conclusion, we have measured the 6MWT distance in dialysis patients with low comorbidity and found the median distance to linearly decrease from 600 m below the 6^th^ decade to 420 m in the 8^th^ decade of life. We found body fat mass and serum TIBC to be the main potentially modifiable predictors of the 6MWT in dialysis population. Of the markers associated with malnutrition-inflammation syndrome, serum TIBC was superior to CRP and albumin in prediction of walked distance. Living with renal failure is a condition not only to independently lower the 6MWT result but it also increases the perceived level of dyspnea at physical exertion. Although hemoglobin concentration was not associated with test performance, it independently negatively predicted perceived shortness of breath and fatigue even at levels in the target range for ESRD population. Interventional studies to improve the physical performance of dialysis patients through modification of these variables are now needed.

## Supporting Information

S1 File Raw study data file. (XLS)

S1 Table Adjusted analysis for prediction of 6MWT in the subsample of dialysis patients. (DOCX)

S2 Table Adjusted analysis for prediction of 6MWT in control subjects. (DOCX)

S3 Table Logistic regression model of the probability of having a more than mild shortness of breath (dyspnea Borg scale grade 3 or more) excluding the lowest quartile of dialysis patients with hemoglobin below 110 g/l. (DOCX)

S4 Table Logistic regression model for the probability of having a rise in dyspnea grade above the median rise of 2 scores. (DOCX)
