# Supplementary material for: Six-Minute Walk Test in Renal Failure Patients: Representative Results, Performance Analysis and Perceived Dyspnea Predictors
Source: PLoS One. 2016 Mar 16;11(3):e0150414. doi: 10.1371/journal.pone.0150414 (PMC4794199; doi:10.1371/journal.pone.0150414)
Supplement: S1 Table — (DOCX) [file pone.0150414.s002.docx]

**S1 Table. Adjusted analysis for prediction of 6MWT in the subsample of dialysis patients.**

| Parameter | Adjusted analysis* | | | |
| --- | --- | --- | --- | --- |
|  | B (SE) | 95% C.I. for B | Partial Eta^2^ | p |
| Age (years) | -2.6 (0.6) | -3.7 to -1.5 | 0.22 | <0.001 |
| Sex (female) | 23.6 (28.8) | -33.6 to 80.9 | 0.008 | 0.41 |
| Body height (cm) | 3.1 (1.3) | 0.6 to 5.5 | 0.07 | 0.02 |
| Spontaneous gait speed (m/s) | 127.1 (40.4) | 46.7 to 207.6 | 0.11 | 0.002 |
| Lean tissue mass (kg) | 0.4 (1.4) | -2.4 to 3.1 | 0.001 | 0.79 |
| Fat tissue mass (kg) | -2.5 (0.9) | -4.3 to -0.8 | 0.09 | 0.005 |
| Davies comorbidity grade 0 vs.2 | 49.8 (32.2) | -14.3 to 113.9 | 0.03 | 0.13 |
| Davies comorbidity grade 1 vs.2 | 13.3 (32.6) | -51.6 to 78.1 | 0.002 | 0.69 |

*N=90, model's R^2^=0.66, adjusted R^2^=0.62, intercept 13.1 m. General linear model, ANOVA.
