# Supplementary material for: Six-Minute Walk Test in Renal Failure Patients: Representative Results, Performance Analysis and Perceived Dyspnea Predictors
Source: PLoS One. 2016 Mar 16;11(3):e0150414. doi: 10.1371/journal.pone.0150414 (PMC4794199; doi:10.1371/journal.pone.0150414)
Supplement: S2 Table — (DOCX) [file pone.0150414.s003.docx]

**S2 Table. Adjusted analysis for prediction of 6MWT in control subjects.**

| Parameter | Adjusted analysis* | | | |
| --- | --- | --- | --- | --- |
|  | B (SE) | 95% C.I. for B | Partial Eta^2^ | p |
| Age (years) | -1.7 (0.5) | -2.6 to -0.8 | 0.09 | <0.001 |
| Sex (female) | 14.9 (42.1) | -68.4 to 98.3 | 0.001 | 0.72 |
| Body height (cm) | 3.6 (1.1) | 1.6 to 5.7 | 0.08 | 0.001 |
| Spontaneous gait speed (m/s) | 70.5 (27.4) | 16.3 to 124.7 | 0.05 | 0.01 |
| Lean tissue mass (kg) | 0.2 (1.1) | -2.1 to 2.4 | 0 | 0.89 |
| Fat tissue mass (kg) | -3.7 (0.6) | -5 to -2.5 | 0.21 | <0.001 |
| Davies comorbidity grade 0 vs.1 | 5.8 (25.7) | -45.2 to 56.7 | 0 | 0.82 |

*N=140, model's R^2^=0.57, adjusted R2=0.54, intercept 84.2 m.
