# Supplementary material for: Six-Minute Walk Test in Renal Failure Patients: Representative Results, Performance Analysis and Perceived Dyspnea Predictors
Source: PLoS One. 2016 Mar 16;11(3):e0150414. doi: 10.1371/journal.pone.0150414 (PMC4794199; doi:10.1371/journal.pone.0150414)
Supplement: S3 Table — (DOCX) [file pone.0150414.s004.docx]

**S3 Table. Logistic regression model of the probability of having a more than mild shortness of breath (dyspnea Borg scale grade 3 or more) excluding the lowest quartile of dialysis patients with hemoglobin below 110 g/l.**

| Parameter | 95% C.I. for odds ratio | | | p |
| --- | --- | --- | --- | --- |
|  | Lower | Odds ratio | Upper |  |
| Age (years) | 0.97 | 1 | 1.02 | 0.88 |
| Spontaneous gait speed (m/s) | 0.03 | 0.14 | 0.7 | 0.02 |
| Rate-pressure product/1000 | 1.08 | 1.16 | 1.25 | <0.001 |
| Hemoglobin (g/l) | 0.92 | 0.96 | 0.99 | 0.02 |
| Fat tissue mass (kg) | 0.96 | 0.99 | 1.03 | 0.74 |
| Body height (cm) | 0.99 | 1.03 | 1.08 | 0.18 |
| Over-hydration (l) | 0.68 | 0.93 | 1.27 | 0.64 |
| Davies comorbidity grade 2 vs.0 | 0.07 | 0.73 | 7.61 | 0.79 |
| Davies comorbidity grade 1 vs.0 | 0.06 | 0.61 | 6 | 0.68 |
| Dialysis dependence | 1.04 | 2.8 | 7.8 | 0.04 |

N=178, model R^2^=0.19 (Nagelkerke), model χ^2^=31.5, p<0.001.
