# Supplementary material for: Six-Minute Walk Test in Renal Failure Patients: Representative Results, Performance Analysis and Perceived Dyspnea Predictors
Source: PLoS One. 2016 Mar 16;11(3):e0150414. doi: 10.1371/journal.pone.0150414 (PMC4794199; doi:10.1371/journal.pone.0150414)
Supplement: S4 Table — (DOCX) [file pone.0150414.s005.docx]

**S4 Table. Logistic regression model for the probability of having a rise in dyspnea grade above the median rise of 2 scores.**

| Parameter | 95% C.I. for odds ratio | | | p |
| --- | --- | --- | --- | --- |
|  | Lower | Odds ratio | Upper |  |
| Age (years) | 0.97 | 1 | 1.02 | 0.88 |
| Spontaneous gait speed (m/s) | 0.02 | 0.08 | 0.39 | 0.002 |
| Rate-pressure product/1000 | 1.05 | 1.13 | 1.2 | <0.001 |
| Hemoglobin (g/l) | 0.93 | 0.96 | 0.99 | 0.003 |
| Fat tissue mass (kg) | 0.96 | 0.99 | 1.02 | 0.49 |
| Body height (cm) | 0.98 | 1.02 | 1.07 | 0.18 |
| Over-hydration (l) | 0.67 | 0.9 | 1.2 | 0.46 |
| Davies comorbidity grade 2 vs.0 | 0.1 | 0.78 | 5.87 | 0.81 |
| Davies comorbidity grade 1 vs.0 | 0.1 | 0.79 | 6.3 | 0.82 |
| Dialysis dependence | 0.96 | 2.57 | 6.83 | 0.06 |

N=199, model R2=0.2 (Nagelkerke), model χ^2^=31.5, p<0.001. The median for the rise in Borg dyspnea score was 2 scores.
